# Rethinking Feyerabend: The “Worst Enemy of Science”?

**DOI:** 10.1371/journal.pbio.1001166

**Published:** 2011-10-04

**Authors:** Ian James Kidd

**Affiliations:** Department of Philosophy, Durham University, Durham, United Kingdom

## Abstract

Philosopher Ian James Kidd reviews Feyerabend's *The Tyranny of Science*.

**Figure:**
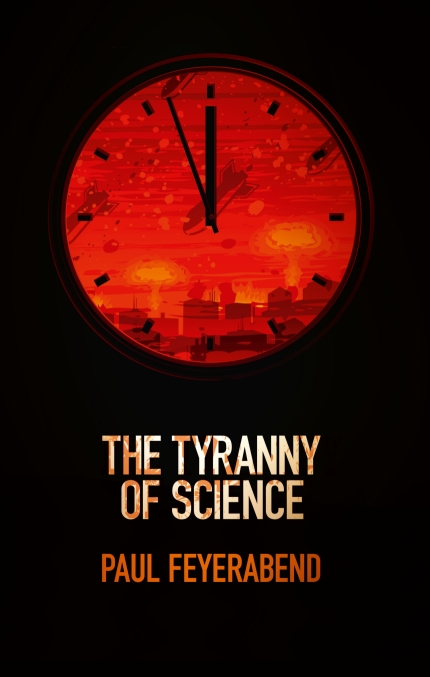
Feyerabend P (2011) The Tyranny of Science. Oberheim E, editor. Cambridge: Polity Press. 180 p. ISBN-13: 978-0745651897 (hardcover). US$54.95

The relationship between science and the philosophy of science is likely to be judged a contested one. Certainly many philosophical debates may seem oblique to the uninitiated (and even then, perhaps still!), whilst recent intellectual debacles have tended to portray philosophers of science in a poor light. During the 1990s, for example, the “Science Wars” erupted over the question of whether scientific theories provided true, objective descriptions of reality, or whether they were simply arbitrary “constructions,” mere mythologies on a par with ancient Greek theogony or medieval magic [1]. There is some truth to such charges, some of it certainly attributable to an unhealthy certain intoxication with trendy theories (like “relativism” and “constructionism”). Yet even if those charges are not always justified, and even if the majority of the philosophy of science is informed and responsible, it remains true that philosophers of science who pitch into debates about the sciences beyond their own professional boundaries must take extra care before letting loose their ideas.

With that proviso in mind, the title of Paul Feyerabend's book, *The Tyranny of Science*, should set off alarm bells, especially since the cover of the book depicts blood-red atomic bombs falling from above onto a desolate city. Indeed, the author himself, who was professor of philosophy at Berkeley and Zurich until his death in 1993, has a “bad reputation” both within and beyond the philosophy of science. Feyerabend was famously dubbed “the worst enemy of science” by *Science*, and even today philosophers of science will tend to associate his name with anti-science polemics, defences of voodoo and astrology, and more besides [2].

Fortunately, Feyerabend is far more sensible than the title and cover of this book and his bad reputation suggest. Although he is reputed as a critic of science, he is not. Feyerabend is critical not of science itself, but of false and misleading images of the sciences. The “tyranny” of the title refers not to an encroaching and disenchanting “scientific worldview,” of the sort popular with some cultural critics, but with the dangers which arose when people fail to understand and appreciate science. Back in the 1960s and early 1970s, Feyerabend urged philosophers of science to take seriously both the history of science and scientific practice—he was a trained physicist himself—and warned his peers that mere abstract reflection on the sciences would produce only idealised fantasies of science, rather than workable models of it. Although subsequent generations of philosophers of science took him seriously, many at the time took his claim as a personal attack—hence the “bad reputation.”

Into the 1980s, Feyerabend began to expand the scope of his ideas. By the beginning of the 1980s, the philosophy of science was a richer discipline, so Feyerabend moved onto new issues. It struck him that public confidence in the sciences was beginning to change into the 1980s. The nuclear accidents at Chernobyl and Three Mile Island, waning interest in the space program, and ambitious new claims on behalf of genetics were beginning to affect public faith in the sciences. Feyerabend was not opposed to such public doubts, but he did worry that the public concerns, although sincere, were too often ill-informed. Worse still, those worries were often amplified by overzealous philosophers who, to his mind, were failing in their job of clarifying concepts, scrutinising arguments, and helping people to articulate and develop their ideas. By the late 1980s, Feyerabend began to take special issue with philosophers who actively encouraged such confusions, for instance by announcing that electrons and genes were mere “social constructions,” or by rebranding forms of relativism, or by implicating “Western Science” in a powerful conspiracy to disempower indigenous cultures—indeed, Feyerabend himself succumbed to such alluring polemics for a time, which partly explains his hostile reaction to them later in his career [3].

Feyerabend's issues with public concerns about science and his worries about philosophers' role in the subsequent debates laid the foundations for the lectures that became *The Tyranny of Science*. In fact, the original title of that lecture series was *Conflict and Harmony*, which is a much better title because it indicates that public engagement with science is dynamic and complex—periods of “conflict” and “harmony,” with scientists, policymakers, philosophers, and other involved groups trying to balance the tensions. Feyerabend's claim here is that many of the conflicts concerning science are based upon confusions about and misperceptions of science—for example, the idea that science is “value-free.” That claim clearly cannot be true, if only because science is necessarily motivated by cognitive and practical values, yet it still features within public and policy debates. Feyerabend's aim in these lectures was to try to demonstrate the science is much more complex than people tend to imagine, and that our thinking about it must be correspondingly complex if we are to make sense of it. Science is only a “tyrant” if we fail to do it justice, and attribute to it exalted characteristics—such as “value-neutrality” or isolation from society—which it lacks.

Throughout his career, Feyerabend defended the claim that there is, in fact, no one thing called “Science,” where that term is understood to refer to something singular and formalised, with uniformly shared methods, theories, and concepts [4]. “Science” as so defined does not exist, even though the idea of it is a powerful one. In its place, urged Feyerabend, we should think and talk about multiple sciences—diverse in their methods and aims, held together by some common values perhaps, but otherwise more an aggregate than the monolith that some writers presume. In order to bring about this reconception of philosophy, Feyerabend urged us to reach out to all the resources at our disposal, a fact evidenced in the eclecticism and immense learning obvious in *Tyranny*. Feyerabend leaps from contemporary social events to the history of geometry, ancient Greek poetry to modern biology, and from the arts to philosophy. The purpose of such intellectual pyrotechnics is not simply to entertain, but to demonstrate just how richly and powerfully the sciences are interlinked with modern human life. For Feyerabend, understanding and appreciation should come as a pair so that, by the end of the lectures, the sciences cease to be the tyrants which contemporary concerns suggest they may be, and which some critics insist they must be.

A key example of the sorts of public worries about science that Feyerabend had in mind concerns genetics. Although human genetic research is conceded to afford wonderful possibilities—for medicine and agriculture, say—there are also corresponding concerns about the abuse of those powers. In the UK, there is a common rhetoric in the popular press concerning “designer babies,” GM crops, “astrological genetics,” and a host of other concerns, each centring upon an implicit worry that the powers of genetic science are too dangerous to be controlled, or that they will be abused. Despite consistent assurances, for instance on the part of the British Government, that genetic research is intensely regulated, public doubts persist. Indeed, the very fact that such doubts exist may frustrate researchers who consider their work to be both morally scrupulous and of clear cognitive and practical value. It may be difficult for those researchers to make willing concessions to public doubts where those doubts are regarded not only as ill-founded, but also as likely to result in further unduly onerous regulation, or even the termination of research projects.

Feyerabend sees a role for philosophers to contribute here. Many worries about genetic research rely upon inarticulate moral or aesthetic concerns—the so-called “yuk factor” which arises at the sight of “Frankenstein” organisms like the famous OncoMouse. In such cases, philosophers can help the public to articulate those concerns and to refine them through argumentation [5]. Often, the worries dissolve upon analysis, and sometimes, of course, are reinforced, but in each case, progress is being made. Feyerabend therefore stressed the need for scientific literacy, philosophical competence, and historical awareness as essential components of informed public engagement with science. Of course, philosophers do not assume a guiding role here; Feyerabend was no fan of the pretensions of some philosophers to resume their ancient, privileged position, but he did consider that their critical sensibilities could be valuable to those wider debates. And since public concerns about the sciences invoke not only scientific facts, but also philosophical judgements about value, purpose, and meaning (the idea of the “sanctity of life,” for instance, demands philosophical input, if only because most of the persons who invoke it are not generally after a biological formulation of it). As long as philosophers remain informed about the sciences they engage with, they can be valuable aids to the project of facilitating public engagement with science—and today, few sciences arouse more fascination, hope, and alarm than the biological sciences [6].

Feyerabend clearly sets himself a broad remit and an ambitious aim. Public concern with the sciences is a persistent and perhaps increasing feature of modern societies. For sure, some of that concern is justified, but much of it is not, for instance because it rests upon false ideas, misperceptions of the science, or because the public imagination has been warped by charged rhetoric and imagery. Feyerabend regretted such misunderstandings and thought that philosophers had an important role to play in helping the public make sense of its concerns. If that sounds paternalistic, it should not—for one thing, philosophers often share those same worries, and for another, philosophers can lay legitimate claim to intellectual skills well-suited to the task of making sense of concerns of science. Feyerabend does not propose that philosophers will pontificate to the public, because he was alert to the fact that philosophers can become “tyrannous” if they, too, cease being engaged with, and responsive to, the concerns and curiosities of the public.

*The Tyranny of Science* should therefore be interpreted as Feyerabend's attempts to dissolve conflicts and establish harmony between science, society, and philosophy, on the one hand, and between scientists, philosophers, and the public, on the other. The concerns and alarms that concerned Feyerabend are not the exclusive preserve of any of those domains—scientific, public, or philosophical—and to properly understand and address them each must cooperate with the other. Tyranny only arises when one of those would try to dominate the others, and Feyerabend's book offers an engaging and entertaining case against such tyranny.

**Editors' note:** Does the cultural divide between science and the humanities, first articulated by C. P. Snow over 50 years ago, still exist between biology and philosophy? In a mini experiment to find out, we asked a philosopher and biologist to review the recent English translation of *Tyranny of Science*, by 20th century philosopher Paul Feyerabend, perhaps best known for rejecting the claim that science is a singular discipline unified by common methods and concepts.

**About the Author**

Ian James Kidd is a Lecturer in Philosophy at the University of Durham and works mainly in the history and philosophy of science. His recent research explores the role of contingency, pluralism, and epistemic virtues in scientific enquiry and has been published in a range of journals in the philosophy of science. Further information can be found on his website at http://www.dur.ac.uk/i.j.kidd/.
